# Supplementary material for: Fast Maximum Likelihood Estimation via Equilibrium Expectation for Large Network Data
Source: Sci Rep. 2018 Jul 31;8:11509. doi: 10.1038/s41598-018-29725-8 (PMC6068132; doi:10.1038/s41598-018-29725-8)
Supplement: Supplementary file 1 — Supplementary File [file 41598_2018_29725_MOESM1_ESM.pdf]

Supplementary Information

Fast Maximum Likelihood Estimation via Equilibrium Expectation for Large Network Data

Maksym Byshkin[1], Alex Stivala[2,1], Antonietta Mira[1,3], Garry Robins[4,2], and Alessandro Lomi[1,4,*]

[1]Università della Svizzera italiana, Institute of Computational Science, Lugano, 6900, Switzerland
[2]Swinburne University of Technology, Centre for Transformative Innovation, Hawthorn Victoria 3122, Australia
[3]Università dell'Insubria, Dipartimento di Scienza e Alta Tecnologia, Como, 22100, Italy
[4]University of Melbourne, School of Psychological Sciences, Parkville Victoria 3010, Australia
[*]alessandro.lomi@usi.ch

Computational details

Pseudocode for the EE algorithm (including technical details of the adaptive method we use to guarantee that the approximate equality (Equation S1) holds), and contrastive divergence (CD) used for initial estimates is detailed as Algorithm S1 and Algorithm S2, respectively. Both these algorithms use an ERGM sampler described in Algorithm S3, however other ERGM samplers may be used, and in particular the IFD sampler[1] was used for the results in the main text.

The step size multipliers $K1_A$ in Algorithm S2 may be obtained from the derivative $\partial \Delta z_A(x_{obs}, \boldsymbol{\theta})/\partial \theta_A$. These derivatives may be approximated by finite differences or as detailed in Algorithm S2. In the estimation results reported in this paper, the number of steps $M1$ was of the order of $10N/m$ where N is the number of network nodes and we have used $m = 1000$ throughout. This number of steps was smaller than that required for the S algorithm to converge, but it was enough to obtain a good starting point for the EE algorithm.

For the EE algorithm, the number of steps M should be large enough for $\theta_A(t)$ to converge. In the estimation results reported in this paper M was of the order of $1000N/m$. A possible choice of K_A constants was suggested in the main text. For the estimation results reported we used a better choice of K_A values. We observed that larger K_A values result in larger fluctuations of $\theta_A(t)$ and faster convergence. Fluctuations may be measured by $\mathrm{sd}(\theta_A(t))/|\overline{\theta_A(t)}|$. We also observe that convergence of the algorithm is faster if fluctuations of different parameters have close values, that is, if $\mathrm{sd}(\theta_A(t))/|\overline{\theta_A(t)}| \approx c_2$ for all A. We thus adapted the values of K_A so that, for all A,

$$\mathrm{sd}(\theta_A(t)) \approx c_2 \cdot \max\{|\overline{\theta_A(t)}|, c_1\} \tag{S1}$$

where we introduce a small positive constant c_1 to avoid singularities. The following constants were used: $c_1 = 0.01$, $c_2 = 10^{-4}$. Larger values of c_2 are also possible. To estimate model parameters on the *Livemocha* network data (Fig. 3 of the main article) we first performed 2.5×10^6 steps with larger K_A values so that $c_2 = 10^{-3}$, and later steps were performed with smaller K_A values so that $c_2 = 10^{-4}$. This approach allows (i) to speed up convergence, and (ii) to check that the estimation results do not depend on the algorithm constants.

In the algorithm descriptions, vectors such as $\boldsymbol{\theta}$, $\boldsymbol{z}$, and $\boldsymbol{dz}$ have dimension equal to the number of model parameters, s. All vector operations are element-wise, e.g. $\boldsymbol{dz}^2$ is the vector consisting of the square of each element of $\boldsymbol{dz}$ and $\boldsymbol{D} \cdot \boldsymbol{dz}$ is the element-wise product of $\boldsymbol{D}$ and $\boldsymbol{dz}$ (a vector of the same dimension, s, as both $\boldsymbol{D}$ and $\boldsymbol{dz}$). The values of the algorithm constants are specified in the algorithm description, with recommend ranges noted in the corresponding comments.

Algorithm S1 EE: Estimate ERGM parameters for an observed network using Equilibrium Expectation.

Precondition: x_{obs} is the observed graph, $\boldsymbol{\theta_0}$ is the initial parameter estimate, $\boldsymbol{D_0}$ is the initial derivative estimate.
Postcondition: Returned value $\boldsymbol{\theta_t}$ is the estimated parameter value.

function EE($x_{\text{obs}}, \boldsymbol{\theta_0}, \boldsymbol{D_0}$)
    $K_A \leftarrow 10^{-4}$ ▷ Multiplier of $\boldsymbol{D}$ to get step size multiplier
    $c_1 \leftarrow 10^{-2}$ ▷ Minimum magnitude of $|\bar{\boldsymbol{\theta}}|$ (small positive constant)
    $c_2 \leftarrow 10^{-4}$ ▷ Multiplier of $|\bar{\boldsymbol{\theta}}|/\text{sd}(\boldsymbol{\theta})$ to limit $\boldsymbol{\theta}$ variance (from 10^{-5} to 0.1)
    $p_1 \leftarrow 2$ ▷ Power to raise $\boldsymbol{dz}$ to for step size (square)
    $p_2 \leftarrow 1/2$ ▷ Power to raise $c_2 \cdot |\bar{\boldsymbol{\theta}}|/\text{sd}(\boldsymbol{\theta})$ to for adapting step size (sqrt)
    $M_{\text{outer}} \leftarrow 1000$ ▷ Steps of Algorithm EE (from 10^2 to 10^4)
    $M_{\text{inner}} \leftarrow 100$ ▷ Inner iterations of Algorithm EE (from 50 to 10^4)
    $m \leftarrow 1000$ ▷ Number of sampler iterations
    $t \leftarrow 0$
    $x \leftarrow x_{\text{obs}}$
    $\boldsymbol{D} \leftarrow \boldsymbol{D_0}$
    $\boldsymbol{dz} \leftarrow \boldsymbol{0}$ ▷ Vector of accumulated change statistics
    for $i \leftarrow 1$ to M_{outer} **do**
        for $j \leftarrow 1$ to M_{inner} **do**
            $(\boldsymbol{dzAdd}, \boldsymbol{dzDel}) \leftarrow$ SAMPLER($x, \boldsymbol{\theta_t}, m$, **True**) ▷ Perform moves: x is updated by accepted proposals
            $\boldsymbol{dz} \leftarrow \boldsymbol{dz} + \boldsymbol{dzAdd} - \boldsymbol{dzDel}$ ▷ Accumulate accepted change statistics
            $\boldsymbol{\theta_{t+1}} \leftarrow \boldsymbol{\theta_t} - \text{sign}(\boldsymbol{dz}) \cdot K_A \cdot \boldsymbol{D} \cdot \boldsymbol{dz}^{p_1}$
            $t \leftarrow t + 1$
        end for
        $\boldsymbol{D} \leftarrow \boldsymbol{D} \cdot \left[c_2 \cdot \frac{\max\left(|\overline{\boldsymbol{\theta}_{t-M_{\text{inner}} \le k < t}}|, c_1\right)}{\text{sd}\left(\boldsymbol{\theta}_{t-M_{\text{inner}} \le k < t}\right)} \right]^{p_2}$ ▷ uses mean and sd of $\boldsymbol{\theta}$ values in inner loop
    end for
    return $\boldsymbol{\theta_t}$
end function

Algorithm S2 Contrastive Divergence for ERGM initial parameter and derivative estimation.

Precondition: x_{obs} is the observed graph.
Postcondition: Return values $\boldsymbol{\theta_t}$ is CD-1 initial parameter estimate and $\boldsymbol{D}$ is initial derivative estimate to use in EE Algorithm.

function CD(x_{obs})
    $K1_A \leftarrow 0.1$ ▷ Multiplier of $\boldsymbol{da}$ to get step size multiplier
    $M1 \leftarrow 50$ ▷ Steps of Algorithm S
    $m \leftarrow 1000$ ▷ Number of sampler iterations
    $x \leftarrow x_{\text{obs}}$
    $\boldsymbol{\theta_0} \leftarrow \boldsymbol{0}$
    $\boldsymbol{D} \leftarrow \boldsymbol{0}$
    for $t \leftarrow 0$ to $M1$-1 **do**
        $(\boldsymbol{dzAdd}, \boldsymbol{dzDel}) \leftarrow$ SAMPLER($x, \boldsymbol{\theta_t}, m$, **False**) ▷ Do not perform moves: x is unchanged
        $\boldsymbol{dz} \leftarrow \boldsymbol{dzAdd} - \boldsymbol{dzDel}$
        $\boldsymbol{dzsum} \leftarrow \boldsymbol{dzAdd} + \boldsymbol{dzDel}$
        $\boldsymbol{D} \leftarrow \boldsymbol{D} + \boldsymbol{dz}^2$ ▷ Approximate expectation of square of change statistics
        $\boldsymbol{da} \leftarrow K1_A / \boldsymbol{dzsum}^2$
        $\boldsymbol{\theta_{t+1}} \leftarrow \boldsymbol{\theta_t} - \text{sign}(\boldsymbol{dz}) \cdot \boldsymbol{da} \cdot \boldsymbol{dz}^2$
    end for
    $\boldsymbol{D} \leftarrow m/\boldsymbol{D}$
    return $(\boldsymbol{\theta_t}, \boldsymbol{D})$
end function

Algorithm S3 Sampler: Sample from ERGM distributions with Metropolis-Hastings using the "basic" sampler, where the proposal is to toggle the edge between two distinct nodes chosen uniformly at random.

Precondition: x is a simple graph, $\boldsymbol{\theta}$ is vector of parameters, m is number of sampler iterations.

Postcondition: Return value $(\boldsymbol{dzAdd}, \boldsymbol{dzDel})$ accumulated change statistics of accepted (add, delete) moves. The graph x is updated by the accepted moves only if *doMove* is True.

```
function SAMPLER(x, θ, m, doMove)
    dzAdd ← 0
    dzDel ← 0
    for s ← 1 to m do
        Choose two nodes i, j (i ≠ j) uniformly at random
        Compute change statistic dz_A for add (if x_ij = 0) or delete (if x_ij = 1) for each statistic A
        α ← min{1, exp(∑_A θ_A · dz_A)}             ▷ ERGM proposal acceptance probability
        if Unif(0,1) < α then                       ▷ Accept change with probability α
            if x_ij = 1 then
                dzDel ← dzDel − dz
            else
                dzAdd ← dzAdd + dz
            end if
            if doMove then
                if x_ij = 1 then
                    x_ij ← 0
                else
                    x_ij ← 1
                end if
            end if
        end if
    end for
    return (dzAdd, dzDel)
end function
```

Proof of equation (12)

Claim. *For any x and A,*

$$\frac{\partial \Delta z_A(x, \boldsymbol{\theta})}{\partial \theta_A} \geq 0.$$

Recall, from equation (5) in the paper, that:

$$\Delta z_A(x, \boldsymbol{\theta}) = \sum_{x'} P\left(x \to x', \boldsymbol{\theta}\right)\left[z_A\left(x'\right) - z_A(x)\right] \tag{S2}$$

Proof. Inserting equation (3) [acceptance probability] from the main text into the expression for the transition probability $P(x \to x', \boldsymbol{\theta}) = q(x \to x')\alpha(x \to x', \boldsymbol{\theta})$ we have:

$$P(x \to x', \boldsymbol{\theta}) = \begin{cases} q(x \to x') & \text{if } \dfrac{q(x' \to x)\pi(x', \boldsymbol{\theta})}{q(x \to x')\pi(x, \boldsymbol{\theta})} > 1 \\ q(x' \to x)\exp\left(\sum_A \theta_A \left[z_A(x') - z_A(x)\right]\right) & \text{otherwise} \end{cases}$$

$$\frac{\partial P(x \to x', \boldsymbol{\theta})}{\partial \theta_A}\left[z_A(x') - z_A(x)\right] = \begin{cases} 0 & \text{if } \dfrac{q(x' \to x)\pi(x', \boldsymbol{\theta})}{q(x \to x')\pi(x, \boldsymbol{\theta})} > 1 \\ q(x' \to x)\left[z_A(x') - z_A(x)\right]^2 \exp\left(\sum_A \theta_A \left[z_A(x') - z_A(x)\right]\right) & \text{otherwise} \end{cases}$$

From (5) in the main text:

$$\frac{\partial \Delta z_A(x, \boldsymbol{\theta})}{\partial \theta_A} = \sum_{x'} \frac{\partial P(x \to x', \boldsymbol{\theta})}{\partial \theta_A}\left[z_A(x') - z_A(x)\right]$$

$$\forall x', \quad \frac{\partial P(x \to x', \boldsymbol{\theta})}{\partial \theta_A}\left[z_A(x') - z_A(x)\right] \geq 0 \implies \frac{\partial \Delta z_A(x, \boldsymbol{\theta})}{\partial \theta_A} \geq 0$$

□

Network statistics typical for social networks

Different types of statistics $z_A(x)$ may be needed to represent different networks. However, a small set of common structural features are present in a large variety of empirical networks (Snijders *et al.*[2]). In the models for undirected networks specified and estimated in the main article, we incorporate some of these statistics that we describe below.

Edge (L)

One of the statistics used for ERGMs is simply the count of the ties (edges) contained in the network x. Denote it by

$$z_L(x) = \sum_{i,j} x_{ij}$$

where $x_{ij} = 1$ if there is a tie between nodes i and j and $x_{ij} = 0$ otherwise.

Isolates

Isolates is simply the count of nodes with no incident edges.

Activity (ρ)

Different nodes may have different attributes. Social network researchers are often interested in how these attributes influence the tendency of actors to form ties. In the main article "activity" and "interaction" statistics are used for a network with binary attributes $a_i = \{0,1\}$. Activity measures the increased propensity for a node with attribute $a_i = 1$ to form a tie, regardless of the attribute of the other node. It is defined as

$$z_\rho(x) = \sum_{i,j} a_i x_{ij}$$

Interaction (ρ_B)

Interaction measures the increased propensity for a node i with $a_i = 1$ to form a tie to another node j also with $a_j = 1$. It is defined as:

$$z_{\rho_B}(x) = \sum_{i,j} x_{ij} a_i a_j$$

Matching

Nodes may also be associate with categorical attribute c_i. The "matching" statistic measures the increased propensity for two nodes to form a tie between them if they have the same value of the categorical attribute. It is defined as

$$z_{\text{Match}}(x) = \sum_{i,j} x_{ij} \delta_{c_i,c_j}$$

where δ is the Kronecker delta function.

Star, two-path and triangle statistics

Other basic statistics are the 2-star count, 3-star count,..., k-star count and triangle count. A k-star is a configuration in which one node is connected to k other nodes (Fig. S1), while a triangle is a complete subgraph of 3 nodes i, j, k so that $x_{ij} = x_{ik} = x_{kj} = 1$. Robins *et al.*[3] and Snijders *et al.*[2] suggested a more general specification. The following configurations were introduced: a k-2-path is a subnetwork comprising 2 nodes, i and j, and a set of exactly k different nodes, sharing ties with both node i and node j: a k-triangle may be defined as such a k-2-path in which nodes i and j are connected by a tie $x_{ij} = 1$ (Fig. S1). Snijders *et al.*[2] also introduced a statistic to model all k-stars with a single parameter. Denote the number of k-stars in the network by $S_k(x)$, the number of k-2-paths by $U_k(x)$ and the number of k-triangles by $T_k(x)$. Then the following statistics are defined.

Alternating stars (AS)

$$z_{AS}(x) = \sum_{k=2}^{N-1} (-1)^k \frac{S_k(x)}{\lambda^{k-2}}$$

Alternating two-paths (A2P)

$$z_{A2P}(x) = U_1(x) - \frac{2U_2(x)}{\lambda} + \sum_{k=3}^{N-2} \left(\frac{-1}{\lambda}\right)^{k-1} U_k(x)$$

Alternating triangles (AT)

$$z_{AT}(x) = 3T_1(x) + \sum_{k=1}^{N-3} (-1)^k \frac{T_{k+1}(x)}{\lambda^k}$$

In the above, $\lambda > 1$ is the geometric weighting parameter, with higher values implying increased chances of higher degree nodes (for alternating k-star). We used $\lambda = 2$ throughout.

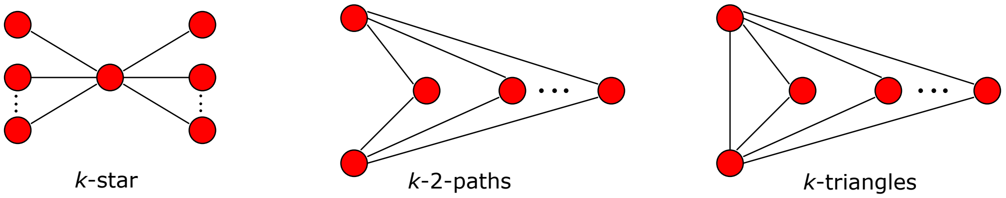

Figure S1. Sub-network configurations: k-stars, k-two-paths and k-triangles.

Network	Number of samples	Number of waves	Number of seeds
A. thaliana PPI	20	3	20
Yeast PPI	20	3	5
Human PPI	20	3	5
C. elegans PPI	20	3	5
Drosophila optic medulla	20	3	2

Table S1. Snowball sampling parameters for the biological networks.

Biological networks

Molecular interactions in living organisms are often viewed as networks[4]. Some are inherently undirected, such as protein-protein interaction (PPI) networks, in which the nodes represent proteins and an edge represents observed binding between two proteins in a particular biological context[5]. Others may be directed, such as gene regulatory networks, where nodes represent operons (contiguous genes which are transcribed as a unit) and edges represent transcriptional interactions between them. That is, a directed edge from operon x to operon y means that x encodes a transcription factor which regulates the expression of y.[6]

We use the new EE method, as well as stochastic approximation via the method of moments with both the IFD[1] and typical ("basic") MCMC samplers[7,8], and snowball sampling with conditional estimation[9,10] to estimate ERGM parameters for six biological networks (four PPI networks, a regulatory network, and a neural network).

The four PPI networks are: an *Arabidopsis thaliana* PPI network[11,12], a yeast PPI network[13] from the Nexus network repository[14], a human PPI network[15], and a *Caenorhabditis elegans* PPI network[16]. Self-loops and multiple edges, where present, were removed.

The *A. thaliana* network has the proteins annotated with various properties as described in the Supporting Online Material of Arabidopsis Interactome Mapping Consortium[11]. In particular we make use of the following protein binary attributes:

Plant-specific Genes defined as plant-specific, absent from other eukaryotic lineages (Supporting Online Material of Arabidopsis Interactome Mapping Consortium[11]).

Kinase Kinase enzymatic activities predicted[17].

Phosphorylated Proteins experimentally shown to be phosphorylated[18].

And the following categorical attributes:

Ubiquitin E by domain Ubiquitin activating E1 enzyme, ubiquitin conjugating E2 enzyme, or ubiquitin ligase E3 enzyme. Domain assignments based on sequence alignments (Supporting Online Material of Arabidopsis Interactome Mapping Consortium[11]). An NA value (which does not match any class) is assigned to other proteins.

Kinase/Phosphorylated Constructed from the Kinase and Phosphorylated binary attributes. Every protein with the Kinase binary attribute is assigned to the Kinase class, and proteins with the Phosphorylated attribute (which do not also have the Kinase attribute) are assigned to the Phosphorylated class, and other proteins are assigned NA.

The *E. coli* regulatory network[6,19] was obtained via the statnet package[20,21]. As in Saul & Filkov[22] and Hummel *et al.* [23], we treat this network as undirected.

As an example of a neural network, a *Drosophila* optic medulla synaptic network[24], found via the ICON[25] database, was obtained from Open Connectome (`http://openconnecto.me/graph-services/download/`)[26]. The nodes in this network represent neurons, with the edges representing synaptic interactions. Here we treat this network as undirected.

Simple structural models, consisting only of the Edge (L), Alternating k-star (AS), Alternating k-triangle (AT), and Isolates parameters were estimated by four methods: the new EE algorithm (with the IFD sampler in the MCMC step), stochastic approximation (SA) via the method of moments (using both the basic and IFD samplers), and snowball sampling with conditional estimation. The latter method first requires that snowball samples are obtained from the network, which are then estimated independently (in parallel) by conditional estimation, and then a point estimate and bootstrap standard errors are estimated as described in Stivala *et al.* [10].

For all methods, 20 estimations are made in parallel (20 snowball samples for snowball sampling, 20 estimations of the full network for the other methods). The snowball sampling parameters used are shown in Table S1.

Table S2 shows the average estimation time and elapsed ("wall clock") time for each network using each method. It is clear that the EE algorithm is able to estimate network parameters much faster than the other methods, taking at most 11 minutes elapsed time, even for networks that take many hours with other methods.

Method	Network	Average sample size	N_c	Avg. estim. time (m)	Elapsed time
EE (IFD sampler)	*A. thaliana* PPI	2160	20	1.1	01 m 50 s
EE (IFD sampler)	Yeast PPI	2617	20	6.6	09 m 07 s
EE (IFD sampler)	Human PPI	4303	20	7.6	10 m 49 s
EE (IFD sampler)	*C. elegans* PPI	5038	20	6.8	09 m 35 s
EE (IFD sampler)	*E. coli* regulatory	418	20	0.6	00 m 43 s
EE (IFD sampler)	*Drosophila* optic medulla	1781	20	4.3	06 m 22 s
SA (IFD sampler)	*A. thaliana* PPI	2160	20	9.2	0 h 34 m 02 s
SA (IFD sampler)	Human PPI	4303	20	49.2	2 h 46 m 54 s
SA (IFD sampler)	Yeast PPI	2617	20	45.6	2 h 02 m 38 s
SA (IFD sampler)	*C. elegans* PPI	5038	20	766.5	25 h 07 m 44 s
SA (IFD sampler)	*E. coli* regulatory	418	20	0.0	0 h 00 m 06 s
SA (IFD sampler)	*Drosophila* optic medulla	1781	20	824.6	72 h 40 m 00 s
SA (basic sampler)	*A. thaliana* PPI	2160	0	—	(time limit)
SA (basic sampler)	Yeast PPI	2617	0	—	(time limit)
SA (basic sampler)	Human PPI	4303	0	—	(time limit)
SA (basic sampler)	*C. elegans* PPI	5038	3	204.5	7 h 40 m 20 s
SA (basic sampler)	*E. coli* regulatory	418	20	1.1	0 h 04 m 06 s
SA (basic sampler)	*Drosophila* optic medulla	1781	0	—	(time limit)
Snowball sampling	*A. thaliana* PPI	490.6	19	26.3	2 h 08 m 24 s
Snowball sampling	Yeast PPI	264.8	19	30.2	3 h 40 m 34 s
Snowball sampling	Human PPI	822.5	18	47.0	3 h 50 m 27 s
Snowball sampling	*C. elegans* PPI	496.4	16	270.7	40 h 00 m 33 s
Snowball sampling	*Drosophila* optic medulla	649.7	15	118.0	7 h 22 m 48 s

Table S2. Average single sample (or full network when snowball sampling not used) estimation time and total elapsed time, using 20 Intel Haswell compute cores (2.3 GHz) on a Lenovo NeXtScale x86 cluster system. The maximum elapsed time limit was set to 99 hours. Average estimation times are over 20 snowball samples (one per core) when snowball sampling is used, or 20 parallel runs (one per core) of the whole network when snowball sampling is not used. N_c is the number of estimations which converged. The *E. coli* regulatory network was not estimated with snowball sampling as it is too small.

The estimated model parameters for all six networks, estimated with all four methods, are shown in Table S3. Although it is many times faster, the EE algorithm obtains estimates that are consistent with, actually almost exactly equal to, those from the MCMCMLE methods. Snowball sampling, while usually able to detect significant effects, is an approximate method and not an MLE, and occasionally does not find significant effects that the MLE methods do. For example the alternating k-triangle parameter on the *A. thaliana* PPI network is not found to be significant when using snowball sampling, while it is by the full network MLE methods.

These results show a significant positive alternating k-triangle (AT) parameter for all the networks, indicating that the triangle motif is significantly over-represented.

Network	Effect	Estimate (95% C.I.)			
		EE (IFD sampler)	SA (IFD sampler)	SA (basic sampler)	Snowball
A. thaliana PPI	AS	2.33 (2.24,2.42)	2.32 (2.23,2.42)	—	2.88 (1.72,3.10)
	AT	1.28 (1.24,1.31)	1.27 (1.23,1.32)	—	0.00 (−0.01,0.01)
	Edge	−14.99 (−15.01,−14.96)	−14.97	—	−14.76 (−16.26,−13.36)
	Isolates	−7.14 (−7.58,−6.69)	−7.12 (−7.58,−6.66)	—	−10.49 (−11.21,−7.95)
Yeast PPI	AS	−0.05 (−0.10,0.01)	−0.05 (−0.10,0.01)	—	0.56 (−0.48,1.29)
	AT	1.86 (1.81,1.91)	1.86 (1.82,1.90)	—	0.85 (0.18,1.07)
	Edge	−7.76 (−7.81,−7.71)	−7.76	—	−6.57 (−13.42,−4.88)
Human PPI	AS	1.32 (1.29,1.35)	1.32 (1.29,1.35)	—	1.29 (0.67,2.04)
	AT	1.37 (1.35,1.38)	1.37 (1.34,1.39)	—	0.03 (0.02,0.07)
	Edge	−11.77 (−11.82,−11.73)	−11.77	—	−9.04 (−13.21,−7.21)
C. elegans PPI	AS	1.04 (1.01,1.07)	1.04 (1.01,1.07)	1.14 (1.03,1.25)	1.06 (0.83,2.16)
	AT	1.59 (1.58,1.61)	1.59 (1.57,1.61)	1.52 (1.47,1.57)	0.35 (0.19,0.41)
	Edge	−11.03 (−11.08,−10.98)	−10.99	−11.41 (−11.78,−11.04)	−8.82 (−13.40,−7.24)
E. coli regulatory	AS	0.45 (0.32,0.59)	0.44 (0.31,0.57)	0.44 (0.19,0.69)	—
	AT	0.78 (0.64,0.93)	0.79 (0.66,0.92)	0.79 (0.61,0.96)	—
	Edge	−6.55 (−6.63,−6.47)	−6.53	−6.53 (−7.24,−5.82)	—
Drosophila medulla	AS	0.23 (0.17,0.30)	0.24 (0.18,0.30)	—	1.17 (−0.64,1.58)
	AT	1.62 (1.56,1.67)	1.61 (1.57,1.65)	—	1.09 (0.86,1.29)
	Edge	−8.14 (−8.19,−8.09)	−8.16	—	−7.70 (−12.51,−5.12)

Table S3. Parameter estimates for the biological networks. Standard errors for methods other than snowball sampling were computed from the inverse covariance matrix of the simulated network statistics[7].

References

1. Byshkin, M. *et al.* Auxiliary parameter MCMC for exponential random graph models. *J. Stat. Phys.* **165**, 740–754 (2016).
2. Snijders, T. A., Pattison, P. E., Robins, G. L. & Handcock, M. S. New specifications for exponential random graph models. *Sociol. Methodol.* **36**, 99–153 (2006).
3. Robins, G., Snijders, T., Wang, P., Handcock, M. & Pattison, P. Recent developments in exponential random graph (p*) models for social networks. *Soc. Networks* **29**, 192–215 (2007).
4. Winterbach, W., Van Mieghem, P., Reinders, M., Wang, H. & de Ridder, D. Topology of molecular interaction networks. *BMC Syst. Biol.* **7**, 90 (2013).
5. De Las Rivas, J. & Fontanillo, C. Protein–protein interactions essentials: key concepts to building and analyzing interactome networks. *PLOS Comput. Biol.* **6**, e1000807 (2010).
6. Shen-Orr, S. S., Milo, R., Mangan, S. & Alon, U. Network motifs in the transcriptional regulation network of *Escherichia coli*. *Nat. Genet.* **31**, 64 (2002).
7. Snijders, T. A. Markov chain Monte Carlo estimation of exponential random graph models. *J. Soc. Struct.* **3**, 1–40 (2002).
8. Wang, P., Robins, G. & Pattison, P. *PNet: program for the estimation and simulation of p^* exponential random graph models*. Department of Psychology, The University of Melbourne (2009).
9. Pattison, P. E., Robins, G. L., Snijders, T. A. B. & Wang, P. Conditional estimation of exponential random graph models from snowball sampling designs. *J. Math. Psychol.* **57**, 284–296 (2013).
10. Stivala, A. D., Koskinen, J. H., Rolls, D. A., Wang, P. & Robins, G. L. Snowball sampling for estimating exponential random graph models for large networks. *Soc. Networks* **47**, 167–188 (2016).
11. Arabidopsis Interactome Mapping Consortium. Evidence for network evolution in an *Arabidopsis* interactome map. *Science* **333**, 601–607 (2011).
12. Swarbreck, D. *et al.* The *Arabidopsis Information Resource (TAIR)*: gene structure and function annotation. *Nucleic Acids Res.* **36**, D1009–D1014 (2008).
13. von Mering, C. *et al.* Comparative assessment of large-scale data sets of protein–protein interactions. *Nature* **417**, 399–403 (2002).
14. Csardi, G. & Nepusz, T. The igraph software package for complex network research. *InterJournal* **Complex Systems**, 1695 (2006). URL http://igraph.org.
15. Rolland, T. *et al.* A proteome-scale map of the human interactome network. *Cell* **159**, 1212–1226 (2014).
16. Huang, X.-T., Zhu, Y., Chan, L. L. H., Zhao, Z. & Yan, H. An integrative *C. elegans* protein–protein interaction network with reliability assessment based on a probabilistic graphical model. *Mol. BioSyst.* **12**, 85–92 (2016).
17. Gribskov, M. *et al.* PlantsP: a functional genomics database for plant phosphorylation. *Nucleic Acids Res.* **29**, 111–113 (2001).
18. Durek, P. *et al.* PhosPhAt: the *Arabidopsis thaliana* phosphorylation site database. an update. *Nucleic Acids Res.* **38**, D828–D834 (2010).
19. Salgado, H. *et al.* RegulonDB (version 3.2): transcriptional regulation and operon organization in *Escherichia coli* K-12. *Nucleic Acids Res.* **29**, 72–74 (2001).
20. Handcock, M. S. *et al.* statnet: Software tools for the representation, visualization, analysis and simulation of network data. *J. Stat. Softw.* **24**, 1548 (2008). URL http://www.jstatsoft.org/v24/i01.
21. Handcock, M. S. *et al.* *statnet: Software Tools for the Statistical Analysis of Network Data*. The Statnet Project (http://www.statnet.org) (2016). URL CRAN.R-project.org/package=statnet. R package version 2016.9.
22. Saul, Z. M. & Filkov, V. Exploring biological network structure using exponential random graph models. *Bioinformatics* **23**, 2604–2611 (2007).
23. Hummel, R. M., Hunter, D. R. & Handcock, M. S. Improving simulation-based algorithms for fitting ERGMs. *J. Comput. Graph. Stat.* **21**, 920–939 (2012).
24. Takemura, S.-y. *et al.* A visual motion detection circuit suggested by *Drosophila* connectomics. *Nature* **500**, 175–181 (2013).

25. Clauset, A., Tucker, E. & Sainz, M. The Colorado Index of Complex Networks (2016). URL https://icon.colorado.edu.

26. Burns, R. *et al.* The Open Connectome project data cluster: scalable analysis and vision for high-throughput neuroscience. In *Proceedings of the 25th International Conference on Scientific and Statistical Database Management*, 27 (ACM, 2013).
